# The Design of Rare-Earth Giant Magnetostrictive Ultrasonic Transducer and Experimental Study on Its Application of Ultrasonic Surface Strengthening

**DOI:** 10.3390/mi9030098

**Published:** 2018-02-27

**Authors:** Shanxiang Fang, Qinjian Zhang, Huiling Zhao, Jingzhou Yu, Yongchen Chu

**Affiliations:** 1 School of Mechanical, Electronic and Control Engineering, Beijing Jiaotong University, Beijing 100044, China; 16116349@bjtu.edu.cn (S.F.); 15121234@bjtu.edu.cn (H.Z.); 16125979@bjtu.edu.cn (J.Y.); 2 School of Mechanical and Electrical Engineering, Jiangxi University of Science and Technology, Ganzhou 341000, China; 3 Automation School, Beijing University of Posts and Telecommunications, Beijing 100876, China; 4 Technology Center Electric Development Department, CRRC Qingdao Sifang Co., Ltd., Qingdao 266111, China; 13125864@bjtu.edu.cn

**Keywords:** rare-earth, ultrasonic transducer, ultrasonic surface strengthening, roughness, hardness

## Abstract

Ultrasonic transducer based on rare-earth giant magnetostrictive materials was designed in accordance with the technical requirements of ultrasonic surface strengthening. The whole structure of the transducer was designed. Modal analysis is made to get the natural frequency of the compound oscillator. The working frequency of the transducer should be guaranteed at about 15.2 kHz and the composite oscillator should have relatively better vibration mode. The magnetic field of the transducer is well sealed and the transducer will not show obvious magnetic flux leakage phenomenon. Which shows the rationality of structural design. Based on this transducer, the ultrasonic surface strengthening experiment on 40 steel was conducted. The surface roughness and hardness of the parts were analyzed after the experiment. The results show that the surface of the parts reach the mirror surface result after the ultrasonic strengthening. When compared to the previous process, the roughness decreases by about 75%, and the surface hardness increases by more than 20%.

## 1. Introduction

Because the surface quality of parts will directly affect the fatigue life, in order to improve the surface quality and precision, parts often need further processing after finishing. More and more methods have been applied to the surface strengthening processing of the parts in modern times. Among that the most common methods include shot peening, rolling strengthening, and ultrasonic vibration extrusion strengthening, etc. Shot peening is mainly used to improve the fatigue properties of metallic components by introducing compressive residual stress and producing strain-hardening in surface layer. Xiao et al. [1] find that impacting density has obvious influence on the peening effects of random and regular peening patterns. It is often used in the parts that are under high stress condition. Also, the thickness of the workpiece can not be too thin (above 2 mm). Rolling strengthening is a kind of plastic processing method. The smooth roller with high hardness have a direct contact with the surface of the workpiece, making the surface produce micro plastic deformation, and then the surface roughness will be improved. After rolling strengthening, work-hardening, and residual stress caused by the plastic deformation on the surface of the workpiece will improve its fatigue strength. Altenberger et al. [2] analyze the effect of deep-rolling on the stress-controlled low and hige cycle behavior of Ti_6_Al_4_V. The surface of the alloy steel surface is reinforced after rolling strengthening. The hardness of the hardened layer is about 0.1–0.8 mm. The improvement of the parts’ surface quality is significantly limited for the reason that the strengthening layer is shallow.

In recent years, ultrasonic machining has developed rapidly and been used in various fields, including ultrasonic liquid abrasive machining, ultrasonic cleaning, ultrasonic welding, ultrasonic-assisted machining, and ultrasonic lithotripsy which is applied to the medical field. Moreover, ultrasonic surface strengthening technology has been proposed and applied in recent years. Fang et al. [3] propose a processing method that the processing head with the compressive pre-stress and high frequency longitudinal impact force hits the workpiece surface to flatten the micro peak-valley on it. At the same time, Surface plastic deformation occurs and the compressive stress is formed, which can improve the surface quality of the workpiece.

At present, the transducer used in ultrasonic machining is mainly piezoelectric ceramic transducer, but its amplitude is small and it needs an ultrasonic horn to amplify the amplitude, which causes power loss. Clark [4] finds that magnetostrictive coefficient of the binary rare-earth iron compounds is significantly large at room temperature, and the Curie temperature is etremely high. Therefore, it is called the rare-earth giant magnetostrictive material. Among them, the properties of Tb_1-*x*_Dy*_x_*Fe_2_ (0.68 ≤ *x* ≤ 0.73) is optimal, and the commodity number is Terfenol-D. According to the research results of Grunwald [5], Terfenol-D offer very attractive and valuable features. The precise small motions with high energy density and fast control response could be applied as well in some automotive and aerospace applications.

Then, with the continuous improvement of this kind of material in the preparation process, the research and application of rare-earth magnetostrictive transducer has entered a stage of rapid development. Zhu et al. [6] report that the rare-earth giant magnetostrictive transducer was first used in low frequency sonar systems by the United States Navy Department, and its resonant frequency of the coil could reach 2000 Hz. In Japan, Wakiwaka et al. [7] designed a sonar transducer with 8 Terfenol-D rods. The electromechanical coupling coefficient of the transducer is 73%, and the signal of acoustic source reaches 192 dB. Dhilsha et al. [8] designed a low frequency giant magnetostrictive transducer. The rare-earth giant magnetostrictive transducer has the advantages of high power, large amplitude and no overheating failure. Son and Cho [9] have constructed an under water sonar transducer using Terfenol-D rod employing open magnetic circuit in order to improve the heat dissipation problem of sonar transducer using Terfenol-D. The sound radiation power can reach to 200 dB at input power of 650 VA at the resonance frequency. In the United States, Stillesjo et al. [10] introduce that Etrema has developed CU18A magnetostrictive transducer whose working frequency is 15~20 kHz, the output amplitude is 6~10 µm, and the interior is able to withstand 150 °C which can be applied in ultrasonic welding. Effect of prestress on the dynamic performace of a Terfenol-D transducer has been studied [11]. The dynamic response of the transducer can be explained as the interaction between the frequency dependent AC losses and the mechanical resonance. For solving the eddy current, Wang et al. [12] propose a giant magnetostrictive actuator based on the use of permanent magnet. Permanent magnet is used for driving magnetic field of Terfenol-D and this method avoids effect of temperature that is caused by skin effect and eddy current of solenoid. Karunanidhi et al. [13] design a magnetostrictive actuator with flexure amplifier and a magnetically biased magnetostrictive actuator for application in high frequency flapper–nozzle servo valve. It has satisfactory static and dynamic characteristics for applications in high-speed actuation systems. Sheykholeslami et al. [14] carride out an experimental comparative study between first and second longitudinal modes of vibration in the Terfenol-D transducer. Results show that higher quality factor in the second mode shape and it has lower sensitivity with Young modulus. Moreover, Δ*E* effect and its influence on vibrational behavior of the this transducer have been studied [15]. With this type of transducer, Δ*E* effect leads to a change in the resonance frequency about 4 percent. The performance of the transducer can be affected negatively if this effect is neglected. They continue to propose an approach to design and fabrication of resonant giant magnetostrictive transducer [16]. This approach can make the transducer having high mechanical quality factor and low damping coefficient. Potential applications of the magnetostrictive technology in simple, high energy density structures with fast responses extend over a range of different industries.

Researches show that ultrasonic surface strengthening technology requires large power, high vibration velocity and displacement amplitude under some process conditions. Good stability of the vibration system is important as well. In addition, the ultrasonic transducer is the key to meet these requirements. Some experiments show that the increase of the vibration speed within the allowable range of the system stiffness can improve the processing efficiency. For the piezoelectric transducer, when the vibration amplitude is too large, the amplitude of tool head will fluctuate periodically due to the precision and power limitation of piezoelectric transducer. This can result in slight light and dark stripe on the surface of the workpiece, and the quality of the workpiece surface will decrease. Features of magnetostrictive applications like power density, precision and dynamic performance are excellent catalysts for the implementation of the technology in high volume applications. Terfenol-D have been developed with stable characteristics over a wide range of temperatures and have a high magnetoelastic coefficient [17]. It is hard for rare-earth ultrasonic transducer to lose polarization and become aging for the characters of magnetostrictive material. It is of great help to ultrasonic surface strengthening technology because of the stronger ability to apply electric power for the magnetostrictive material’s large power density and small size and light weight for its low Young’s modulus [18]. So it is worthwhile to use rare-earth giant magnetostrictive materials instead of piezoelectric ceramics to make the transducer that is used in the field of ultrasonic surface strengthening technology.

Therefore, this paper introduces the design and finite element analysis of rare-earth giant magnetostrictive ultrasonic transducer. Additionally, the experimental study on the ultrasonic surface strengthening technology baised on the transducer was conducted.

## 2. The Design of Rare-Earth Giant Magnetostrictive Ultrasonic Transducer

### 2.1. The Structural Design of the Transducer

**Figure 1:**
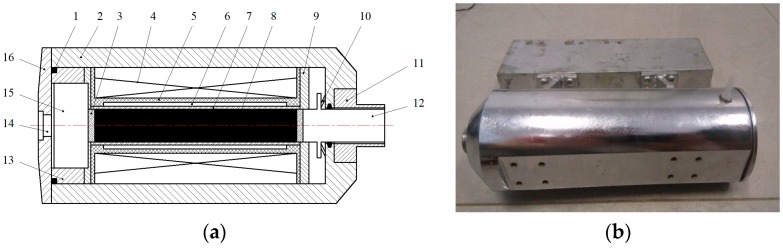
The structure diagram of the transducer. (**a**) Structure diagram. 1-Sealing ring; 2-Case; 3-Permanent magnet; 4-Coil; 5-Bobbin; 6-Cooling water; 7-Sleeve; 8-Terfenol-D rod; 9-Magnetic sheets; 10-Disc spring; 11-Connectors; 12-Output rod; 13-Adjustment ring; 14-Inout port; 15-Balance weight; 16-Back cover; (**b**) The assembled transducer.

Seen from the structure diagram of the transducer in Figure 1, the case of the transducer is cylindrical. The bobbin and the excitation coil wound on the bobbin are coaxially mounted in the case, and the coil provides an alternating magnetic field. Terfenol-D rod is coaxially installed in the center of the exciting coil. Permanent magnets are installed on both ends of the rare earth rod to provide a bias magnetic field. The Terfenol-D rod is supported by the back cover, and the anather end of Terfenol-D rod is connected with the output rod. Back cover, Terfenol-D rod and output rod constitute the compound vibrator which is a key part of the transducer. The sleeve between the bobbin and Terfenol-D rod is made of boron nitride. Boron nitride has high temperature resistance and good heat dissipation, and the surface is smooth so that the resistance of the Terfenol-D rod in the telescopic process will be greatly reduced. The bobbin is made of nylon with a low density and no magnetic permeability. Magnetic sheets is made of silicon steel with larger magnetic permeability. They are installed at both ends of the bobbin to make the magnetic field be closed to minimize magnetic leakage. Which can effectively improve the uniformity of the magnetic field in the solenoid.

### 2.2. The Slicing Design of the Terfenol-D Rod

**Figure 2:**
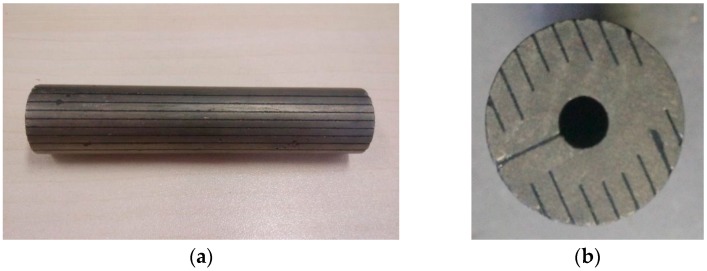
Terfenol-D rod after section treatment. (**a**) Terfenol-D rod; and, (**b**) Section treatment.

Eddy current loss and the heat dissipation of transducer are the key problems of the research. Two measures are taken to reduce the eddy current loss. One is to change the structure of Terfenol-D rod, and the other is to design a set of auxiliary cooling system. Terfenol-D rod is cylindrical before processing. As shown in Figure 2, the middle of the rod is emptied and cut into slices in order to reduce the eddy current effect. Considering that the power and heat of the transducer is large, the water cooling is adopted. As shown in Figure 1a, there is a cavity between the sleeve and the bobbin, with circulating cooling water inside, which can cool the Terfenol-D rod effectively.

### 2.3. The Design of the Compound Vibrator

**Figure 3:**
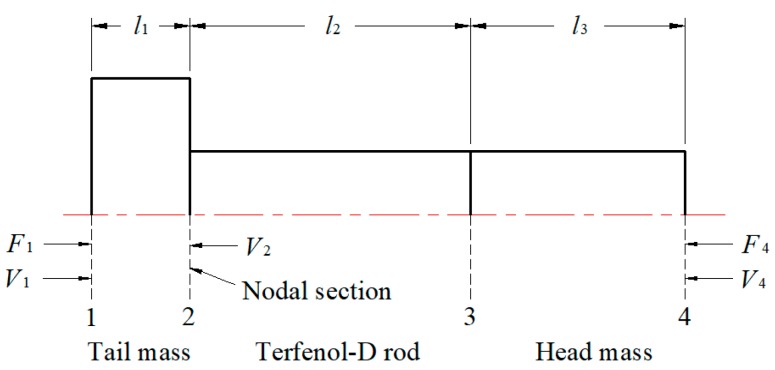
Simplified model of compound vibrator.

In the rare-earth giant magnetostrictive ultrasonic transducer, because the coil does not participate in the vibration, the transducer can simplified as a compound vibrator. Which could be divided into three main parts: excitation part (based on magnetostrictive material: Terfenol-D rod), backing part (tail mass: balance weight) and matching part (head mass: output rod), as shown in Figure 3.

No vibration occurred at the node location, so the compound vibrator can be fixed on the shell through the node section. Both ends of the compound vibrator are free, therefore *F*_1_ = *F*_4_ = 0. The particle velocity was zero at the nodal section, therefore *V*_2_ = 0.

The size of the Terfenol-D rod is Ø20 × 100 mm. According to the 1/4 wavelength theory, the length of the head mass should be equal to the 1/4 of the wavelength of the corresponding sound wave which propagates in it, Equation (1) can be obtained:

(1) $$l_{3}=\frac{\lambda_{3}}{4}$$

where, *l*_3_ is length of the head mass; *λ_3_* is the wavelength of the corresponding sound wave.

The Equation (2) is the frequency equation, as obtained in accordance with the simple harmonic motion and the boundary conditions of the compound vibrator.

(2) $$\tan\left(k_{1}l_{1}\right)\times \tan\left(k_{2}l_{2}\right)=\frac{Z_{2}}{Z_{1}}$$

where *Z* = *ρcS* is the acoustic impedance. *ρ* is the density of the material. *c* is the velocity of sound. *S* is the cross-sectional area. *k* = *ω/c* is the wave number. *ω* = *2πf* is the frequency of harmonic vibration. *f* is the frequency.

In order to ensure that the amplitude of the transducer are mainly concentrated on the output rod, and improve the vibration speed ratio between the front end and back end, the head mass should be made of materials with relatively low acoustic impedance (such as hard aluminum alloy and aluminum-magnesium alloy), and the tail mass should be made of materials with higher acoustic impedance (such as stainless steel and brass). The performance parameters of the selected materials are shown in Table 1.

Equations (1) and (2) could be solved by the certain known parameters substitution (such as the cross section area of each part of compound vibrator, the material parameters that are shown in Table 1 and the size of Terfenol-D rod), therefore the unknown parameters could be acquired: *l*_3_ = 14.4 mm; *l*_1_ = 63.3 mm.

## 3. Finite Element Analysis of Rare-Earth Giant Magnetostrictive Ultrasonic Transducer

The working performance of rare-earth ultrasonic transducer is closely related to the natural frequency of the compound vibrator: excitation part (based on magnetostrictive material: Terfenol-D rod), backing part (tail mass: balance weight), and matching part (head mass: output rod). Only the work frequency of the transducer near the natural frequency of the Terfenol-D, can the output amplitude reach the maximum value. Its working performance is related with the closure and the uniformity of the magnetic field. Therefore, in this paper, modal analysis is made to get the natural frequency of the compound vibrator. The magnetic field transducer structure is analyzed to examine the rationality of the structure and materials.

### 3.1. The Finite Element Analysis of the Composite Oscillator Vibration Modal

The vibration of Terfenol-D rods, output rod and counterweight are concentrated in the axial direction because of their cylindrical structure. So, only the axial vibration can be taken into account when analysing. The water cooling system will provide big cavity for water flowing, which will affect the work frequency of the transducer. The working state of the transducer may decrease significantly at high frequency (usually for more than 20 kHz). So, the performance of the transducer is tested in the frequency range: 10~30 kHz.

**Figure 4:**
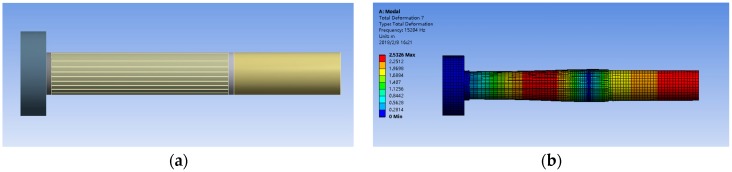
The modal diagrams in the range of 10 to 30 kHz. (**a**) model of compound vibrator; (**b**) The optimal vibratiom mode.

As illustrated in Figure 4a, the slicing-designed The Terfenol-D rod with size of Ø20 × 100 mm was adopted in this paper, central empty with a diameter of 5 mm. There is a permanent magnet at each end of the Terfenol-D, so it is added to the analytical model of the compound vibrator. The boundary conditions was set as that both ends of the compound vibrator were free and the nodal section was fixed. The output rod is made of hard aluminium and the counterweight is made of stainless steel. The parameters of density, elastic modulus, and Poisson’s ratio are set according to Table 1.

From the analysis of Figure 4b, the following conclusion can be achieved: the working frequency of the transducer should be guaranteed at about 15,204 Hz, and the composite oscillator will have relatively better vibration mode, without bending and torsion. This provides a research basis for the study of the later transducer and ultrasonic surface strengthening test.

### 3.2. Finite Element Analysis of the Magnetic Field

The working performance of Terfenol-D depends on the closeness and uniformity of the magnetic field. The closeness and uniformity of the magnetic field depend on the material properties of each part of the transducer (the most important is the permeability of the material). The purpose of magnetic field analysis is to verify that the selection of the material is rational and there is no obviously magnetic leakage phenomenon in the whole structure. This provides the basis for the follow-up production of the transducer.

The impact of the inlet and outlet of water as well as the signal line access are ignored in order to simplified the model of transducer. So, the transducer can be approximated as an axial symmetry model. Two-dimensional geometric model of transducer is established, and the material parameters used in modeling are shown in Table 2.

**Figure 5:**
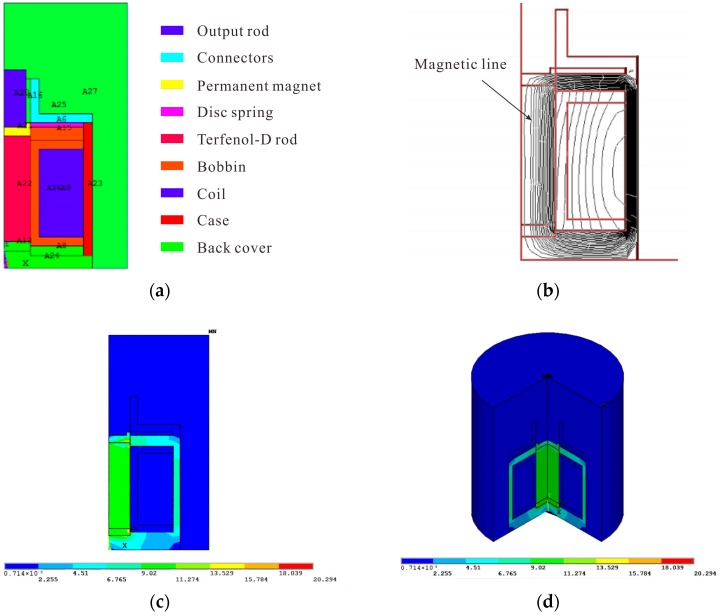
Finite Element Analysis of the Magnetic Field. (**a**) Transducer model; (**b**) Magnetic line of force distribution; (**c**) The magnetic flux density distribution; and, (**d**) The magnetic flux density distribution after expand.

After modeling, different material properties are assigned and each region is numbered, as shown in Figure 5a. Then, the analysis continues with meshing the model and setting the boundary conditions: applying a magnetic lines parallel conditions for model peripheral nodes. And the current density is 6 A/mm^2^.

After calculation, it can be seen from Figure 5b that the magnetic field is well sealed and the transducer does not show obvious magnetic flux leakage phenomenon. The distribution of magnetic line is uniform, which basically meets the magnetic lines closed principle. As can be seen from Figure 5c,d, the magnetic flux density of the entire transducer distributed unevenly. But, the maximum magnetic flux density of the Terfenol-D rod in the middle and the loop circuit basically meets the working requirements of the transducer. So, for it can meet the design requirements of the transducer in general, the design of the magnetic circuit structure and material selection is rational.

### 3.3. Performance Testing of the Rare-Earth Giant Magnetostrictive Transducer

In the actual production process, due to a series of external factors, such as temperature, static and dynamic load, abrasion of transducer, and different workpieces, the natural frequencies of the ultrasonic vibration system will fluctuate in a small range. In order to solve this problem, the ultrasonic driver of the transducer must have the function of automatic frequency tracking. The TH-CC-1 type ultrasonic driver is used in this paper. It has different frequency gears and there is a frequency tracking range at one gear. So the ultrasonic driver can realize the real-time tracking of the optimal resonant frequency of the transducer near the gear. According to the above analysis, these three frequency gears (15 kHz, 20 kHz and 25 kHz) are used to measure the amplitude of a transducer. The vibration amplitude of the ultrasonic transducer under different waveform and frequency is measured and the results are shown in Table 3.

Analysis of results: according to the above test results, it is not difficult to see that when the frequency is the same, the amplitude of the ultrasonic transducer under square wave is the largest, and under the same waveform, as the frequency increases, the amplitude of the transducer has a tendency to decrease. The amplitude reached the maximum at the frequency of 15 kHz under square wave, and this provides the basis for the follow-up ultrasonic surface strengthening test.

The performance of ultrasonic transducers are usually described using the quality factor, which can be obtained in accordance with impedance analysis of the transducer by impedance analyzer. *f*_1_ and *f*_2_ are the frequencies that the displacements of the transducer were attenuated by 3 dB. Δ*f = f*_2_ − *f*_1_ shows bandwidth of the transducer. *f*_1_, *f*_2_ and resonant frequency *f*_s_ are measured by impedance analyzer: *f*_s_ = 15,107.3 Hz; *f*_1_ = 15,034.8 Hz; *f*_2_ = 15,202.4 Hz. The mechanical quality factor *Q_m_* of the transducer can be calculated using Equation (3). The mechanical quality factor of the transducer is not very high, because the bandwidth of the transducer is relatively large. In the process of surface strengthening, the load is constantly changed. If the transducer has large bandwidth, then the phenomenon of detuning and stopping will not occur easily.

(3) $$Q_{m}=\frac{f_{s}}{f_{2}-f_{1}}=\frac{15107.3}{15202.4-15034.8}=90.14$$

The amplitude of this transducer can reach more than 10 μm without ultrasonic horn. It has the advantages of large power, high vibration velocity and good stability of the vibration system. But, there are still some problems to be solved. The magnetic field design of the transducer is very important. In this paper, a permanent magnet with a simple structure is used to provide a bias magnetic field. In the later stage, a better controllable DC circuit can be used to provide the bias magnetic field. The temperature effect of the transducer can be analyzed to guide the improvement of the cooling system. In the market, the ultrasonic drivers are mainly applied to the piezoelectric transducer. The matching degree between driver and magnetostrictive transducer directly affects the output performance. It is difficult to find a suitable ultrasonic driver that matches the magnetostrictive transducer. The development of ultrasonic driver for the magnetostrictive transducer should be studied in the future.

## 4. Experimental Study on the Ultrasonic Surface Strengthening System Based on Rare-Earth Giant Magnetostrictive Ultrasonic Transducer

### 4.1. Test Equipment

**Figure 6:**
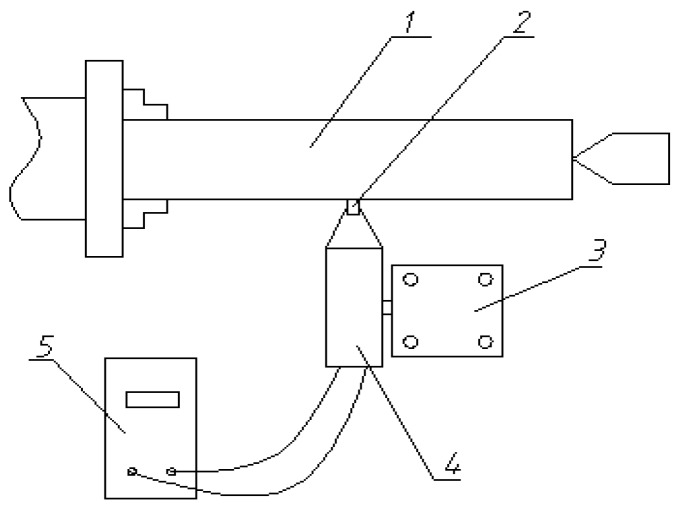
Schematic diagram of the ultrasonic surface strengthening system. 1-Workpiece; 2-Processing head; 3-Tool holder; 4-Trasducer; 5-Ultrasonic power supply.

The ultrasonic strengthening device is clamped on the lathe to be tested, and the connection mode is shown in Figure 6.

The workpiece is fixed on the lathe by three-jaw chuck and lathe center, and the transducer is fixed on the tool holder of the lathe. The power of the transducer is supplied by ultrasonic power source. During the ultrasonic surface strengthening, the workpiece rotates and the transducer drives the processing head to contact and squeeze the workpiece at ultrasonic frequency. Meanwhile, the transducer move left and right with the tool holder to strengthen the surface of the workpiece.

### 4.2. The Experiment Scheme

**Figure 7:**
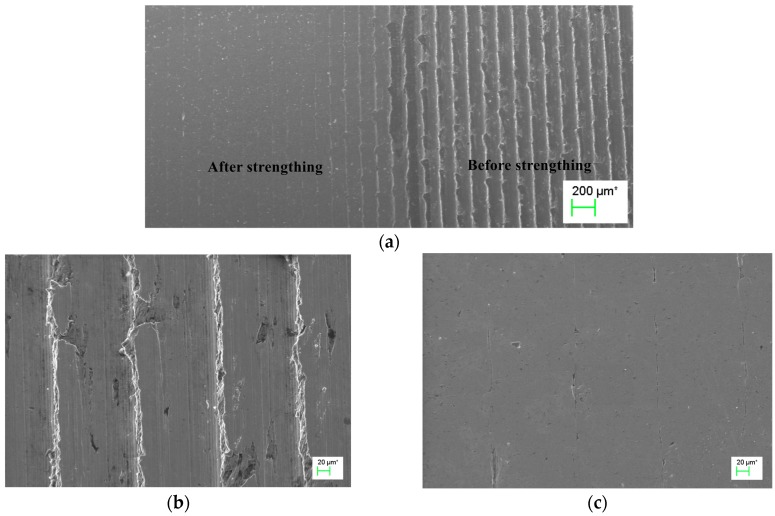
Appearance contrast before and after ultrasonic surface strengthening. (**a**) Appearance contrast before and after strengthening (20×); (**b**) Before strengthing (200×); (**c**) After strengthing (200×).

The material used in this experiment is 40 Steel, which is usually used as the materials for axles of high speed trains. The ultrasonic transducer introduced in the preceding part of this paper was used in this experiment. Gear of the ultrasonic driver for the experiment is 15 kHz. Parameters of ultrasonic surface strengthening mainly include the extrusion variable *P*, rotational speed *v*, feed *f*, processing head radius *r*, ultrasonic frequency, amplitude of the transducer, and so on. Different parameters have verified influences on the results of ultrasonic surface strengthening. The relationship between them is complicated. Therefore, this paper only carry out experimental research on the extrusion variable *P*, rotational speed *v* and feed *f*, and the rest of the parameters are given fixed values. Processing head selects superhard alloy with radius *R* = 5mm. The specific parameters are set in Table 4, and the comparison results for the work-surface appearance before and after ultrasonic surface strengthening are illustrated in Figure 7. It can be seen clearly that the the uneven surface of the workpiece is flatted so that the ultrasonic surface strengthening technology can improve on the surface quality effectively.

### 4.3. Analysis of Experimental Results

#### 4.3.1. Roughness Analysis

The surface roughness of parts has the great influence on the performance of the parts. They are mainly reflected in the following aspects: (1) Effect on friction and wear; (2) Effect on fitting property. Surface roughness will affect the stability of the fitting property; (3) Effect on fatigue strength; and, (4) Effect on contact stiffness. It will affect the working accuracy and vibration resistance of the parts; (5) Effect on corrosion resistance. In addition, surface roughness has a great influence on the sealing performance of the combination surface, appearance quality and surface coating. Therefore, the surface roughness measurement and comparative analysis of the strengthened specimens have been carried out.

This measurement instrument uses the needle contact to measure the surface roughness and profile shape. The natural diamond pin slowly slides along the measured surface, and the curvature radius of the pin is about 2 μm. In this way, the roughness value of the workpiece surface is measured.

**Figure 8:**
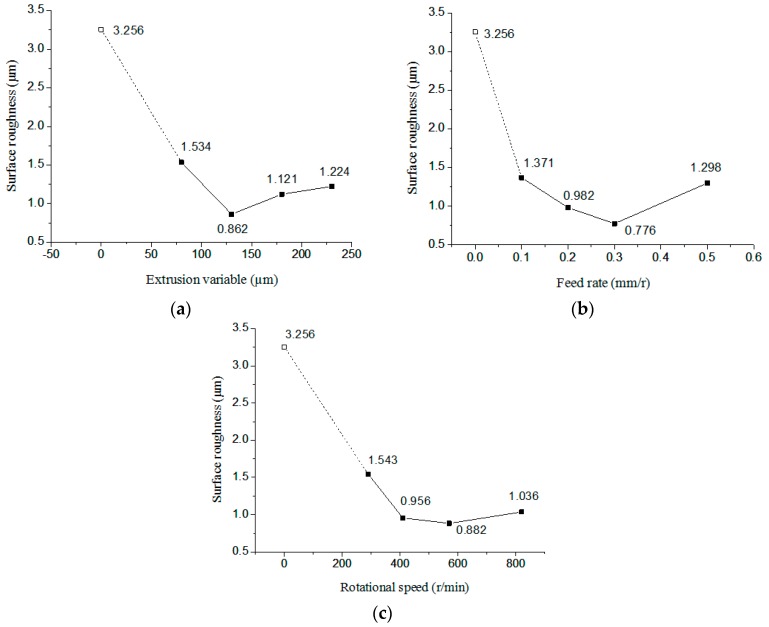
The variation tendency of surface roughness with different parameters. (**a**) Effect of the extrusion variable; (**b**) Effect of the feed rate; (**c**) Effect of the rotational speed.

As shown in Figure 8, extrusion variable, rotational speed, and feed rate have impact on the surface roughness. The roughness of the workpiece surface will change when a single variable parameter is changed. At first, roughness basically decreases, then increases. Therefore, in order to obtain the best surface roughness value for different materials and tool head, reasonable selection of extrusion variable, rotational speed and feed rate is extremely important.

#### 4.3.2. Hardness Analysis

The hardness of the material represents the ability of the material to resist plastic deformation. The surface hardness of the material can reflect the fatigue damage process of the material. There is a corresponding relationship between the fatigue damage process and the change of surface hardness. When the hardness is improved, the stiffness, tensile strength, and impact toughness of parts will be improved as well. Therefore, the hardness measurement and comparative analysis of the strengthened specimens have been carried out.

**Figure 9:**
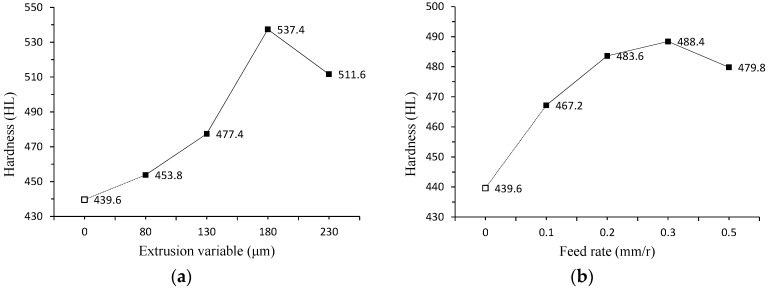

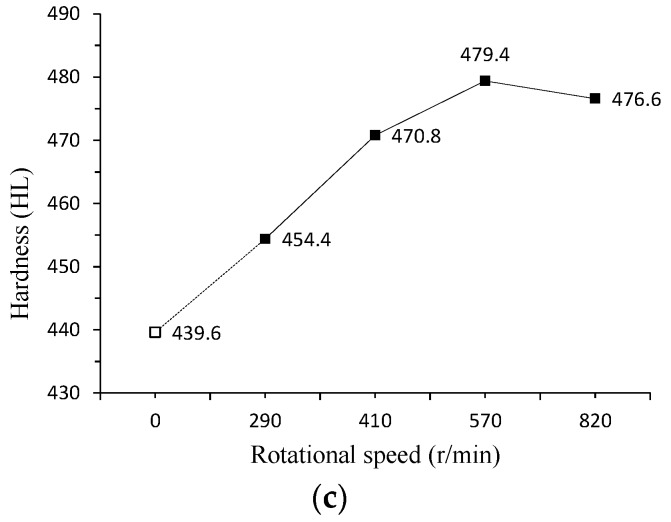
The variation tendency of hardness with different parameters. (**a**) Effect of the extrusion variable; (**b**) Effect of the feed rate; (**c**) Effect of the rotational speed.

Each section is measured for five times and its average is taken. The experimental results are shown in Figure 9. The hardness value of the workpiece with ultrasonic surface strengthening is much higher than the ones that are not strengthened. The hardness values increase at first and then decrease with the increase of the parameter values. Based on the analysis of the data, when the parameter is too large, the depth of the hardening layer decreases, leading to the decrease of the hardness. Also, when the parameter is too small, it can not reach the effect of extrusion strengthening. So, the parameter selection should be moderate.

The internal microstructure of the workpiece was readjusted by ultrasonic surface strengthening. The structure becomes more uniform and compact. The surface hardness is greatly improved with the improvement of the structural strength. When the hardness is improved, the stiffness, tensile strength, and impact toughness of the parts have been improved as well.

In summary, after ultrasonic surface strengthening based on rare-earth giant magnetostrictive ultrasonic transducer, the surface quality of the test-pieces has been significantly improved. The surface roughness can be reduced by about 75% and the hardness can be increased by more than 20%. Therefore, the application of rare-earth giant magnetostrictive ultrasonic transducer in ultrasonic surface strengthening technology is a valuable research direction.

## 5. Conclusions

In this paper, the theoretical design and analysis of the overall structure of the rare-earth giant magnetostrictive ultrasonic transducer are introduced, and the experimental study on the whole ultrasonic system is carried out. Then the roughness and hardness of the specimens are measured and analyzed. The following conclusions can be summarized based on above experimental results:

(1) The working frequency of the transducer should be guaranteed at about 15.2 kHz. The composite oscillator will have relatively better vibration mode, and the output displacement will be much larger.
(2) When the frequency is the same, the amplitude of the ultrasonic transducer under square wave is the largest, and under the same waveform, as the frequency increases, the amplitude of the transducer has a tendency to decrease. The amplitude reached the maximum under the gear of 15 kHz and square wave.
(3) Under the experimental conditions of this paper, the surface of the workpiece can reach the mirror surface, the surface roughness can be reduced by about 75%, and the hardness can be increased by more than 20% after ultrasonic surface strengthening.

These fully prove that the ultrasonic surface strengthening system based on rare-earth giant magnetostrictive materials is a new and promising technology in the field of surface strengthening.

## Figures and Tables

**Table 1:** Material parameter of duralumin, Terfenol-D and stainless Steel.

Material	Density (Kg/m^3^)	Elastic Modulus (GPa)	Poisson Ratio	Acoustic Velocity (m/s)
Stainless steel	7800	195	0.3	4935
Terfenol-D	9250	27.5	0.21	1724
Duralium	2790	71.5	0.34	5056

**Table 2:** Permeability of Different material.

Material	Terfenol-D	Pure Iron	Duralium	Silicon Steel Sheets	NdFeB
Magnetic permeability	5~10	400	1	7000~10,000	1.05

**Table 3:** The vibration amplitude of the ultrasonic transducer under different waveform and frequency.

Waveform	Frequency (kHz)	Amplitude 1 (mm)	Amplitude 2 (mm)	Amplitude 3 (mm)	Average Value (mm)
Cosine wave	15	0.008	0.006	0.008	0.0073
	20	0.006	0.006	0.006	0.006
	25	0.004	0.004	0.004	0.004
Triangular wave	15	0.006	0.006	0.006	0.006
	20	0.006	0.006	0.004	0.0053
	25	0.004	0.004	0.004	0.004
Square wave	15	0.012	0.012	0.010	0.0113
	20	0.010	0.008	0.008	0.0087
	25	0.006	0.008	0.008	0.0073

**Table 4:** Processing parameters of ultrasonic extrusion.

Test-Pieces	Invariants	Variables			
Test-piece 1	Keep the rotational speed n and feed f unchanged *n* = 570 r/min, *f* = 0.2 mm/r	Changing the extrusion variable *P*/μm			
		80	130	180	230
Test-piece 2	Keep the rotational speed n and extrusion variable P unchanged *n* = 570 r/min, *P* = 130 μm	Changing the feed *f*/mm/r			
		0.1	0.2	0.3	0.5
Test-piece 3	Keep the feed f and extrusion variable P unchanged *f* = 0.2 mm/r, *P* = 130 μm	Changing the rotational speed *n*/r/min			
		290	410	570	820
